# New Cocrystals of Antipsychotic Drug Aripiprazole: Decreasing the Dissolution through Cocrystallization

**DOI:** 10.3390/molecules26092414

**Published:** 2021-04-21

**Authors:** Wenwen Liu, Ru Ma, Feifei Liang, Chenxin Duan, Guisen Zhang, Yin Chen, Chao Hao

**Affiliations:** 1 School of Pharmacy, Jiangsu Ocean University, Lianyungang 222005, China; 2019220322@jou.edu.cn (W.L.); 2020000031@jou.edu.cn (R.M.); ylwbs1028@163.com (F.L.); 2019220308@jou.edu.cn (C.D.); gszhang@hust.edu.cn (G.Z.); 2 Department of Biomedical Engineering, College of Life Science and Technology, Huazhong University of Science and Technology, Wuhan 430074, China

**Keywords:** cocrystal, aripiprazole, resveratrol, kaempferol, dissolution rate

## Abstract

Cocrystallization is an important route to tuning the solubility in drugs development, including improving and reducing. Five cocrystals of aripiprazole (ARI) with resveratrol (RSV) and kaempferol (KAE), ARI-RSV, ARI_2_-RSV_1_·MeOH, ARI-KAE, ARI-KAE·EtOH, ARI-KAE·IPA, were synthesized and characterized. The single crystal of ARI_2_-RSV_1_·MeOH, ARI-KAE·EtOH, and ARI-KAE·IPA were analyzed by single crystal X-ray diffraction (SCXRD). The SCXRD showed multiple intermolecular interactions between API and the coformers, including hydrogen bond, halogen bond, and π-π interactions. Dissolution rate of the two nonsolvate ARI-RSV and ARI-KAE cocrystals were investigated through powder dissolution experiment in pH = 4.0 acetate buffer and pH = 6.8 phosphate buffer. The result showed that RSV could reduce the dissolution rate and solubility of ARI in both medium through cocrystallization. However, KAE improved the dissolution rate and solubility of ARI in pH = 4.0 medium, on the contrary, the two solubility indicators of ARI were both reduced for ARI-KAE cocrystal.

## 1. Introduction

Drug cocrystal screening has become a widely used method to improve the physicochemical properties of an active pharmaceutical ingredient (API) [1]. An active pharmaceutical ingredient (API) cocrystallized with another or more cocrystal coformers (CCF) at a certain stoichiometric ratio through hydrogen bonds, van der Waals forces, or other noncovalent interactions, which the multiple components are both in the same crystal lattice, is a cocrystal [2,3,4,5]. Researches on the application performance of drug cocrystals mainly focus on the following aspects: improving drug solubility and dissolution rate [6,7,8,9,10,11,12]; improving drug chemical stability [13,14,15]; improving thermal stability [15,16]; reducing the hygroscopicity [14,17,18,19]; reducing photodegradation [20,21]; improving the tabletability [12,22]; reducing the bitterness [23]; improving the bioavailability [10,11,24,25]; and improving the permeability [26,27,28]. ESTEVE laboratory announced the success of the Phase II clinical trial of tramadol and celecoxib cocrystals for the treatment of acute pain, and subsequently, the review article on drug combination therapy by EMA (European Medicines Agency) made the multidrug cocrystals (MDC) become the superstar [29,30]. MDC may have the potential advantages of synergy, complementary mechanisms, enhancing the solubility and dissolution of at least one component, and improving bioavailability [31,32].

**Figure 1:**
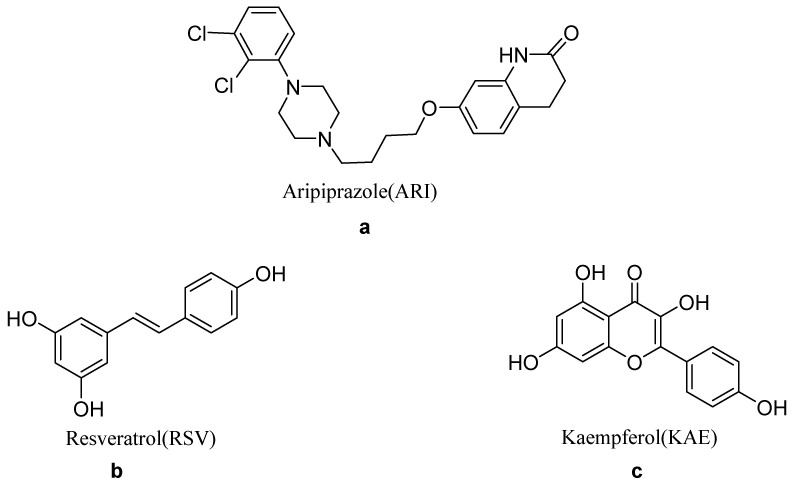
Chemical structures of the aripiprazole (**a**), resveratrol (**b**), and kaempferol (**c**).

Aripiprazole (ARI, Figure 1a) is a third-generation atypical antipsychotic drug [33]. It is a polymorphic compound with more than 10 crystalline forms, including nine anhydrous polymorphs, one hydrate, and four solvates (ethanol, methanol n-propanol, and 1,2-dichlorethane) [34,35,36,37,38,39,40,41]. The equilibrium solubility of ARI in water is 0.00001%, *w*/*v*, which pKa in 20% aqueous ethanol is 7.6 (20% ethanol, at 25 °C) [34]. ARI has been developed into a variety of dosage forms, including tablets, orally disintegrating tablets, oral liquids, intramuscular injections, sustained-release tablets, and digital tablets. Therefore, tuning the solubility and dissolution rate is important for different routes of administration. Recently, cocrystals have been used to change the dissolution and stability of ARI. For example, Nanubolu and Cho reported cocrystals of ARI with a variety of polyphenolic hydroxyl compounds [42,43]. Among them, the maximum ARI concentration from aripiprazole-orcinol (ARI-ORC) was 6.4 mg/L, which was four times greater than pure ARI powder. Zhao published the cocrystals of ARI with organic acids [44]. The six multi-component crystals all show high stability and low hygroscopicity, but their dissolution behaviors were different. In addition, aripiprazole long-acting injection has muscle irritation [45], and reducing muscle irritation is a great significance for improving patient compliance.

Resveratrol (RSV, Figure 1b), a naturally occurring polyphenol, has a variety of functions, including antioxidants, anti-inflammatory, anti-aging, cardioprotective, and neuroprotective activities, and is beneficial to human health. The solubility of RSV in water is 3 mg/100 mL, and the pKa is 9.22. In addition, animal studies have shown that it can penetrate the blood–brain barrier [46]. It is reported that resveratrol produced anti-anxiety and anti-psychotic effects in mouse anxiety and schizophrenia models [47]. The cocrystal of RSV can improve the physical and chemical properties of RSV. In 2016, Zhou reported cocrystals of RSV with 4-amin obenzamide (RSV-4ABZ) and isoniazid (RSV-ISN) [12]. In a wide range of pH, both cocrystals showed higher solubility than pure RSV. In 2017, He reported that RSV cocrystals could enhance powder solubility and increase the tensile strength of tablets under compression pressure, providing guidance for formulation and process development [48]. Kaempferol (KAE, Figure 1c) is a flavonoid compound widely found in vegetables, fruits, and Chinese Medicinal materials, with a slightly solubility in water and a pKa of 6.34 (most acidic, 25 °C). It has various biological functions such as antioxidant, anti-inflammatory, anti-cancer, liver protection, and improving the symptoms of metabolic syndrome. It is safe and non-toxic, and has good development and application prospects [49]. In 2016, He published cocrystals of kaempferol with d/l-proline, which could improve the solubility of pure kaempferol [50].

Yin reported that slow-release cocrystals of oxaliplatin with flavonoids delayed hydrolysis and reduced toxicity [51]. Preparing cocrystal of ARI with RSV and KAE, which have low solubility, expect to reduce the solubility of ARI to achieve the purpose of long-acting treatment. Therefore, it is hoped to form a cocrystal of ARI with RSV and KAE, which have anti-inflammatory effects, to reduce muscle irritation while reducing the solubility of ARI.

In the present work, in order to adjust the solubility and dissolution rate, we choose two insoluble polyphenols, RSV and KAE, which have central nervous system activity, as coformer. The cocrystallization of ARI with RAV and KAE was carried out by the solvent volatilization method, anti-solvent method, and liquid-assisted grinding method. We synthesized five cocrystals of aripiprazole (ARI) with RSV and KAE, ARI-RSV, ARI_2_-RSV_1_·MeOH, ARI-KAE, ARI-KAE·EtOH, ARI-KAE·IPA. We got the single crystal of ARI_2_-RSV_1_·MeOH, ARI-KAE·EtOH, and ARI-KAE·IPA, which are suitable for SCXRD analysis, and their crystallographic characteristics were described in detail. The cocrystal structure was also characterized by powder X-ray diffraction (PXRD), differential scanning calorimetry (DSC), thermogravimetric analysis (TGA), and attenuated total reflection Fourier’s transform infrared spectroscopy (ATR-FTIR). Moreover, the dissolution behavior of two non-solvate cocrystals were studied at different pH medium.

## 2. Results and Discussion

### 2.1. Single Crystal X-ray Diffraction Analysis (SCXRD)

Data of the three cocrystal solvate were collected by SCXRD to investigate the intermolecular interactions between the API and coformer. The corresponding crystallographic data and refinement details are summarized in Table 1, and the hydrogen bonds geometry are listed in Table 2.

**Figure 2:**
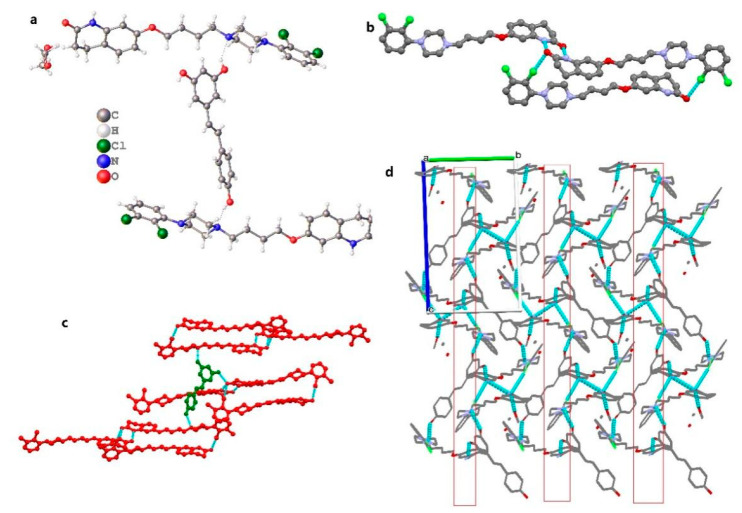
(**a**) The asymmetric unit for ARI_2_-RSV_1_·MeOH (methanol is disorder), (**b**) ARI tri-molecular motif, (**c**) three ARI tri-molecular motif linked to RSV, (**d**) 2D sheet along c axis.

ARI_2_-RSV_1_·MeOH crystallized in the Pn space group. The asymmetric unit contains two aripiprazole molecules, one resveratrol molecule and one methanol solvent molecule, as shown in Figure 2a. Three adjacent aripiprazole molecules form an ARI tri-molecular motif through hydrogen bonds (N3-H3⋯O4 and N6-H6A⋯O2) and halogen bonds (C-Cl⋯O) (Figure 2b). Each resveratrol molecule connects with three ARI tri-molecules motifs through three hydrogen bonds (O5-H5A⋯N5, O6-H6B⋯O4, and O7-H7⋯N) (Figure 2c). Along the ac plane, a layered hydrogen bond network is formed through N-H⋯O, O-H⋯N, O-H⋯O hydrogen bonds and C-Cl⋯O halogen bonds, as shown in Figure 2d.

**Figure 3:**
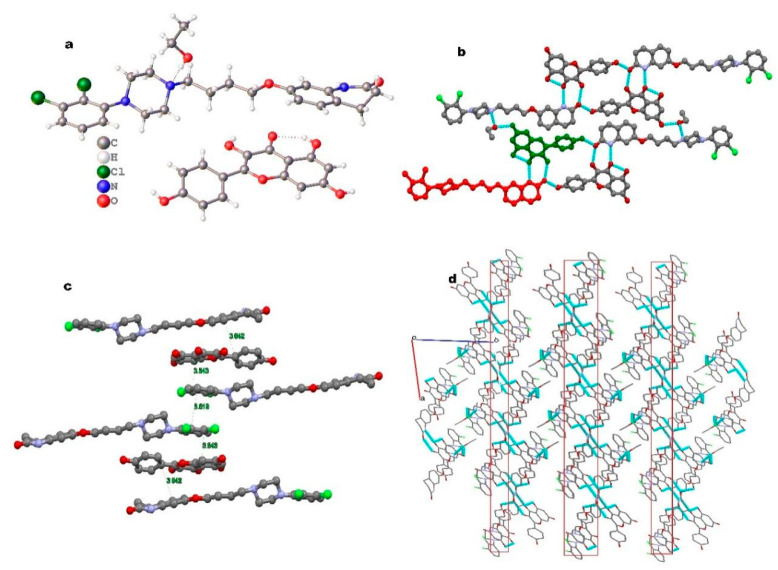
(**a**) The asymmetric unit for ARI-KAE·EtOH, (**b**) hydrogen bonds, (**c**) π-π interaction, (**d**) 2D sheet along [011].

The crystal structure of ARI-KAE·EtOH was in the P-1 space group; the asymmetric unit contains an aripiprazole molecule, a kaempferol molecule, and an ethanol molecule, as shown in Figure 3a. Kaempferol and aripiprazole form dimers through N1-H1⋯O4 and O6-H6⋯O9 hydrogen bonds, and the dimers form tetramers through O2-H2A⋯O9 hydrogen bonds, and the tetramers motif were connected by two molecules ethanol through the O7-H7A⋯N3 hydrogen bond with aripiprazole and the O3-H3⋯O7 with kaempferol, which form a 1D hydrogen bond chain along the a-axis (Figure 3b). Figure 3c shows the π-π interaction along the [110] crystal plane; the π-π interaction is formed between benzene ring of the 4-hydroxyphenyl on kaempferol and the benzene ring of dihydroquinolinone on aripiprazole, the distance is about 3.54 Å; another two type π-π interactions are formed, the benzene ring of the dichlorophenyl group on aripiprazole contacted with the benzene rings of the dichlorophenyl group on the another aripiprazole and the benzene ring of the benzopyran group on kaempferol, and the interaction distances are about 3.82 Å and 3.54 Å, respectively. Figure 3d shows the stacking structure of the 2D layer hydrogen bond network along the [011] crystal plane.

**Figure 4:**
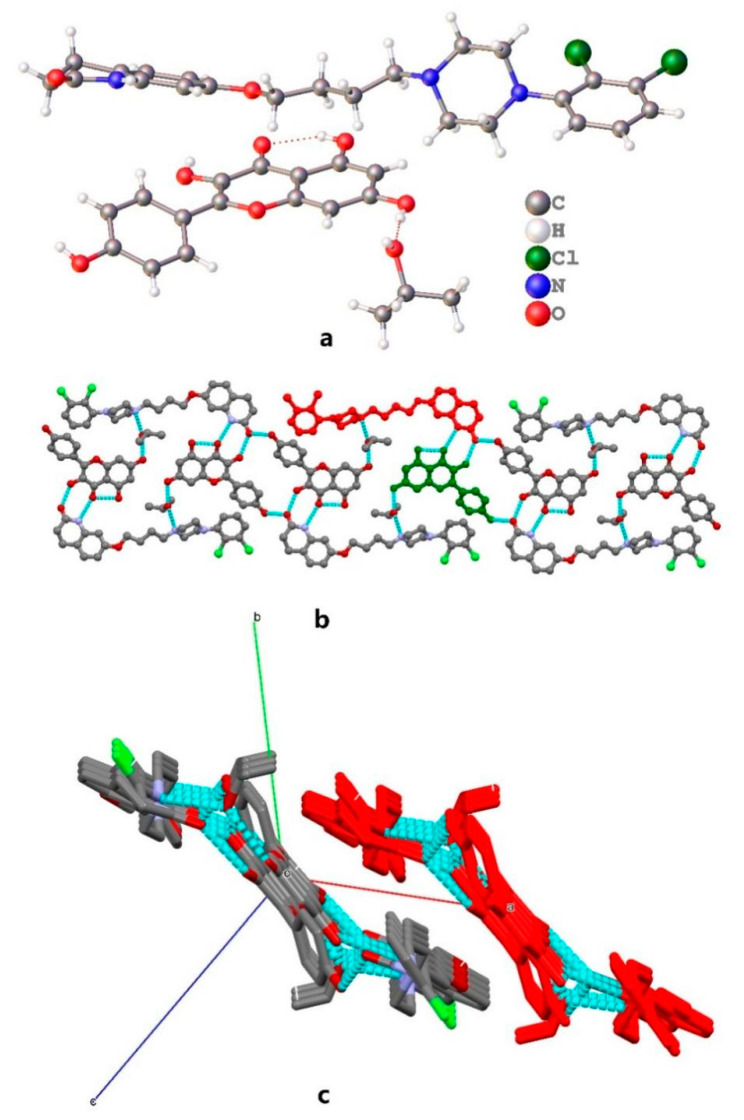
(**a**) The asymmetric unit for ARI-KAE·EtOH, (**b**) hydrogen bonds, (**c**) two parallel hydrogen bonds chain along [111] direction.

ARI-KAE·IPA crystal structure also belongs to P-1 space group; the asymmetric unit contains an aripiprazole molecule, a kaempferol molecule and an isopropanol molecule, as shown in Figure 4a. Kaempferol and aripiprazole form a dimer through N3-H3⋯O6 and O7-H7⋯O2 hydrogen bonds, and the dimers forms a tetramer through O8-H8⋯O2 hydrogen bonds. The tetramers were connected by two isopropanol molecules through O9-H9⋯N2 and O3-H3A⋯O9 hydrogen bonds, forming a 1D hydrogen bond chain along the [111] direction (Figure 4b). The 1D hydrogen bond chain packing together through π-π interactions and other weak intermolecular interactions, formed a binary parallel hydrogen bond chain along the [111] direction (Figure 4c).

Both ARI-KAE·EtOH and ARI-KAE·IPA, the solvent participated in the hydrogen bonding at the hydrogen bond chain between aripiprazole and kaempferol. It may be that the solvent is more conducive to improving the stability of the crystal.

### 2.2. Powder X-ray Diffraction Analysis (PXRD)

**Figure 5:**
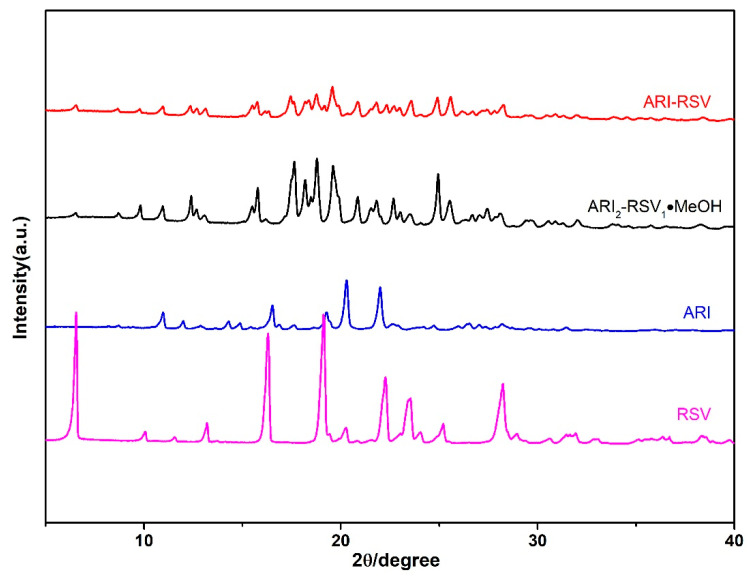
The PXRD pattern if ARI and RSV cocrystals.

**Figure 6:**
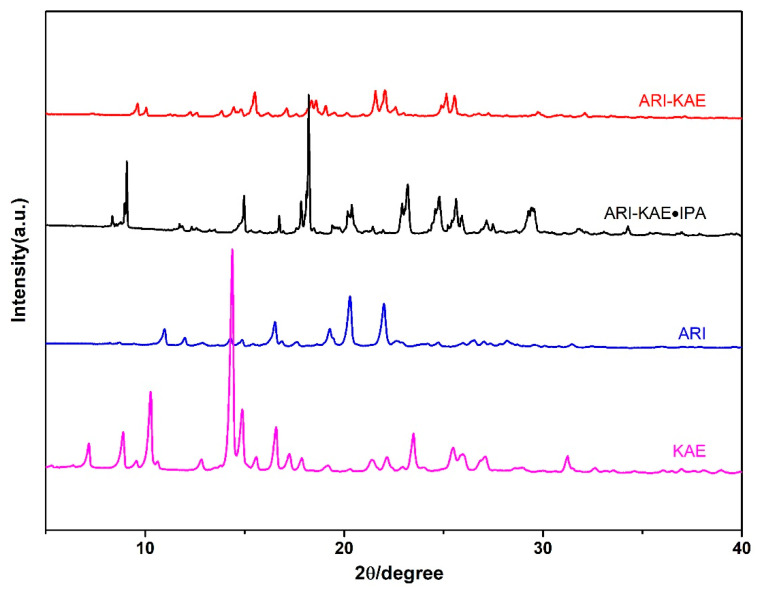
The PXRD pattern if ARI and KAE cocrystals.

Powder X-ray diffraction analysis is a powerful tool to determine whether a cocrystal is formed or not. Therefore, the powder X-ray diffraction characterization of monomer components and drug cocrystal is performed (Figure 5 and Figure 6). The characteristic peaks of ARI are at 10.93°, 11.96°, 14.31°, 14.85°, 16.51°, 19.26°, 20.26°, 22.03°; the characteristic peaks of RSV are at 6.55°, 10.06°, 13.20°, 16.31°, 20.21°, 22.24°, 25.20°; while the characteristic peaks of ARI-RSV are at 6.51°, 8.64°, 9.79°, 10.94°, 12.32°, 15.78°, 19.56°, 20.87°, 24.89°; the characteristic diffraction peaks of ARI_2_-RSV_1_·MeOH are at 6.55°, 8.75°, 9.83°, 10.95°, 12.40°, 15.80°, 17.64°, 18.85°, 19.61°, 24.94°.

Figure 6 shows that the characteristic peaks of KAE are at 7.17°, 8.87°, 10.25°, 14.39°, 16.60°, 23.49°. The characteristic peaks of ARI-KAE are at 9.58°, 10.06°, 15.50°, 18.35°, 18.60°, 19.09°, 21.58°, 22.07°, 25.14°, 25.55°; the characteristic peaks of ARI-KAE-IPA are at 8.35°, 9.07°, 14.97°, 16.74°, 18.21°, 23.17°. Experimental and calculated PXRD patterns of cocrystals are available in Supplementary Materials.

### 2.3. Thermal Analysis

**Figure 7:**
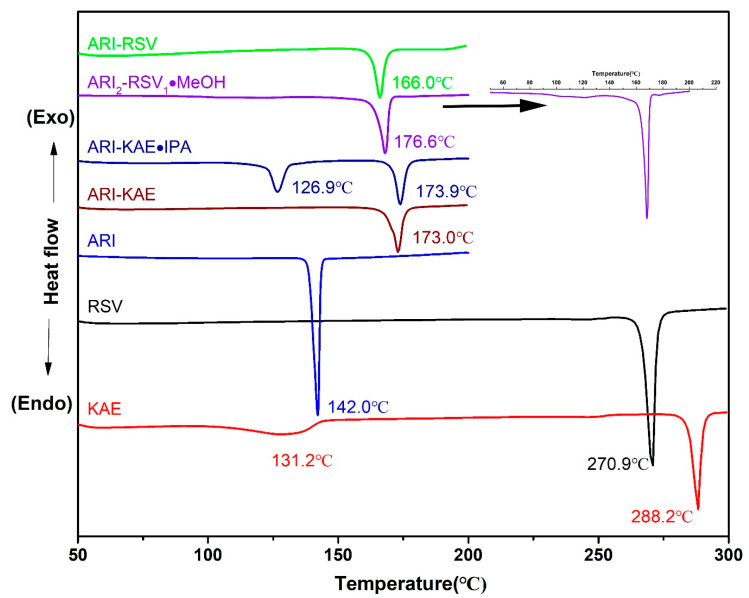
DSC curves of the pharmaceutical cocrystals.

**Figure 8:**
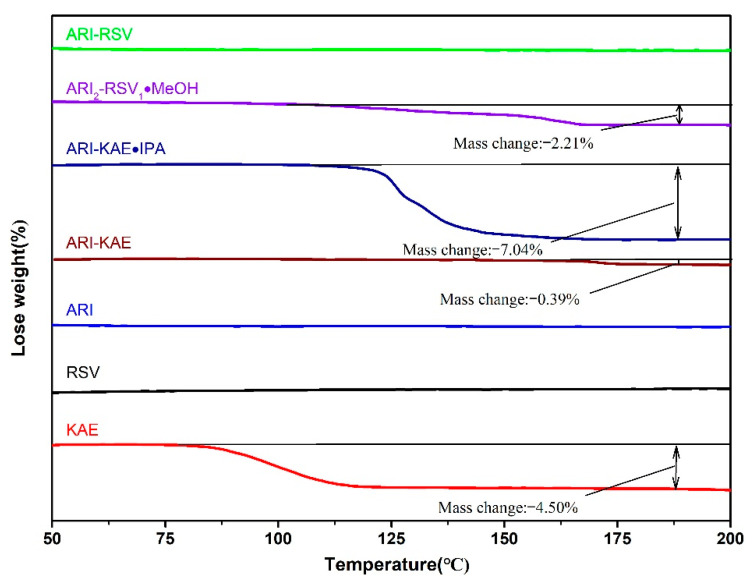
TGA curves of the pharmaceutical cocrystals.

In order to analyze the thermal behavior of each monomer components and drug cocrystal, DSC and TGA analyses were performed, the result is shown in Figure 7 and Figure 8. The endothermic peak of pure ARI is at 142 °C, which corresponds to aripiprazole polymorph Form II. The melting endothermic peak of RSV is at 270.9 °C. KAE shows two endothermic events at 131.2 °C and 288.2 °C, respectively. The broad endothermic peak at about 131.2 °C should be caused by dehydration, which corresponds to the 4.5% weight loss in TGA before melting. The melting endothermic peak of ARI-RSV is a single peak at 166 °C, indicating that a cocrystal is formed instead of a physical mixture. ARI_2_-RSV_1_·MeOH shows two endothermic events at 120 °C (the inserted small image) and 176.6 °C, the endothermic peak at 120 °C is caused by the partial liberation of methanol, which corresponds to the 2.21% (theoretical value is 2.8%) weight loss in TGA; 176.6 °C is the melting endothermic peak of the cocrystal. ARI-KAE also shows a single endothermic peak at 173 °C, indicating that a cocrystal was formed. There are two endothermic peaks for ARI-KAE-IPA, at 126.9 °C and 173.9 °C; the endothermic peak at 126.9 °C is caused by the removal of one molecule of isopropanol, which corresponds to 7.04% (theoretical value is 7.6%) weight loss at the range of 110 °C to 180 °C in TGA. The melting point of each cocrystal is basically between the melting points of API and coformers.

### 2.4. Attenuated Total Reflection Fourier’s Transform Infrared Spectroscopy (ATR-FTIR)

**Figure 9:**
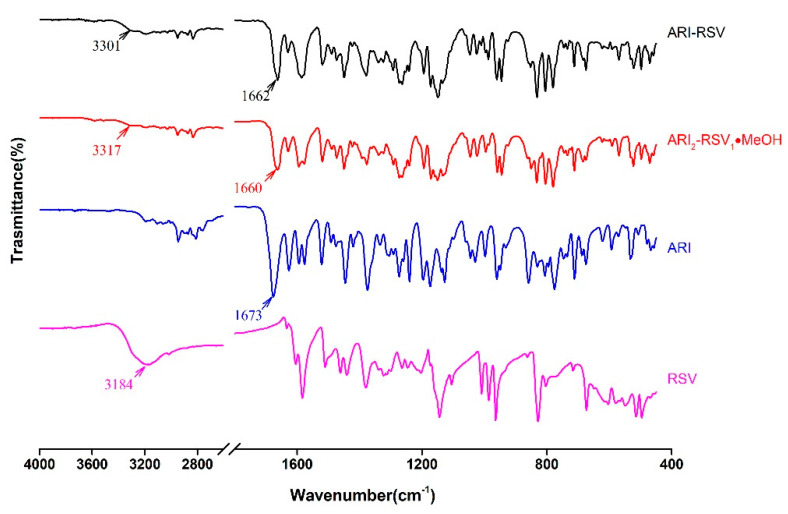
The ATR-FTIR spectrum of ARI, RSV, and their cocrystals.

**Figure 10:**
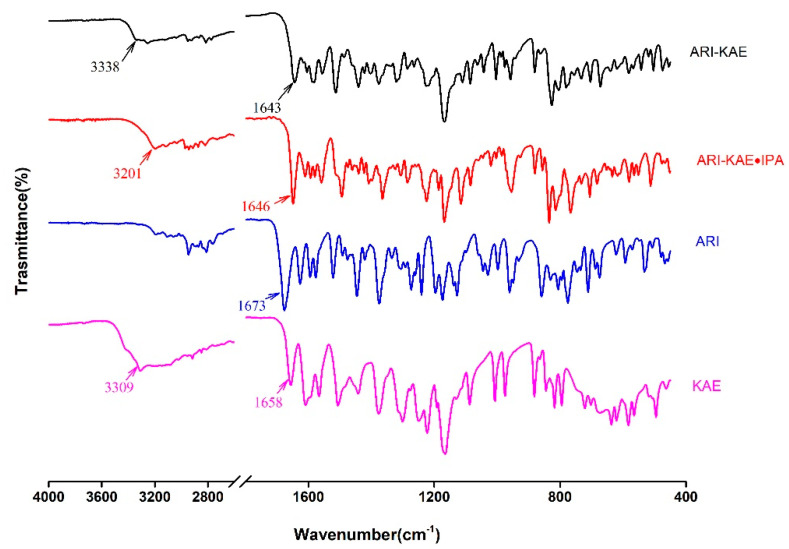
The ATR-FTIR spectrum of ARI, KAE, and their cocrystals.

ATR-FTIR is fast and nondestructive characterization methods for cocrystals. The infrared spectrum curves of API and drug cocrystals are shown in Figure 9 and Figure 10. In cocrystals, the presence of hydrogen bonds averages the density of the electron cloud, so the vibration frequency of C=O decreases. The stretching vibration peak of C=O of ARI occurs at 1673 cm^−1^, and the stretching vibration peak of C=O of KAE occurs at 1658 cm^−1^. The stretching vibration peak of C=O has changed in cocrystal, ARI_2_-RSV_1_·MeOH at 1660 cm^−1^, ARI-RSV at 1662 cm^−1^, ARI-KAE at 1643 cm^−1^, and ARI-KAE·IPA at 1646 cm^−1^. The stretching vibration peak of -OH of RSV occurs at 3184 cm^−1^, KAE occurs at 3309 cm^−1^. In the cocrystal, -OH has a stretching vibration peak change due to hydrogen bond association, such as ARI_2_-RSV_1_·MeOH at 3317 cm^−1^, ARI-RSV at 3301 cm^−1^, ARI-KAE at 3338 cm^−1^, and ARI-KAE·IPA at 3201 cm^−1^.

### 2.5. Dissolution Rate

**Figure 11:**
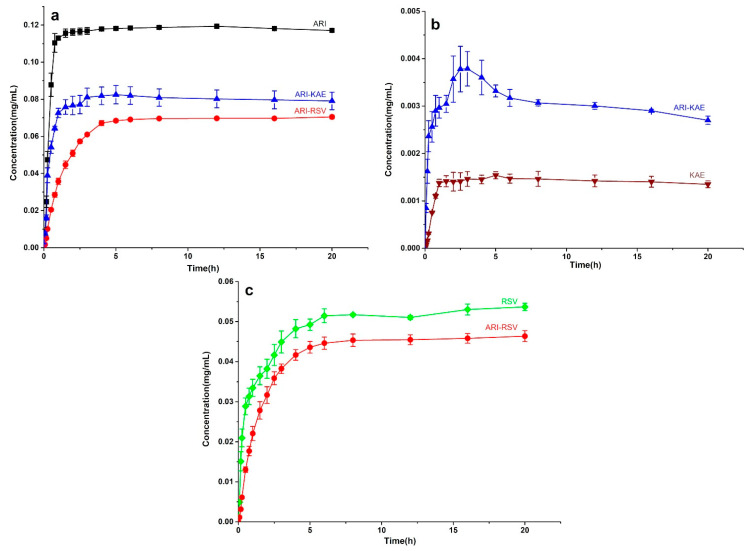
The dissolution profile of ARI-KAE and ARI-RSV at pH = 4.0 acetate buffer, (**a**) ARI from pure ARI, ARI-RSV, and ARI-KAE, (**b**) KAE from pure KAE and ARI-KAE, (**c**) RSV from pure RSV and ARI-RSV.

The dissolution curves of ARI, RSV, KAE, and their cocrystal in pH = 4.0 acetate buffer is shown in Figure 11. As exhibited in Figure 11a, both ARI-RSV and ARI-KAE reduced the dissolution rate and solubility of ARI in the pH = 4.0 medium. The solubility of ARI in ARI-KAE decreased for about 33%, and to ARI-RSV it reduced for about 42%. Since the dissolution of ARI is pH-dependent, the solubility of ARI at pH = 4.0 was significantly improved (120 μg/mL); however, it did not improve the dissolution of KAE in a positive correlation compared with at pH = 6.8. (Figure 11b). Due to the mutual dissolution inhibition effect of ARI and RSV, the dissolution of RSV in ARI-RSV was also significantly inhibited, which was reduced for about 20%.

**Figure 12:**
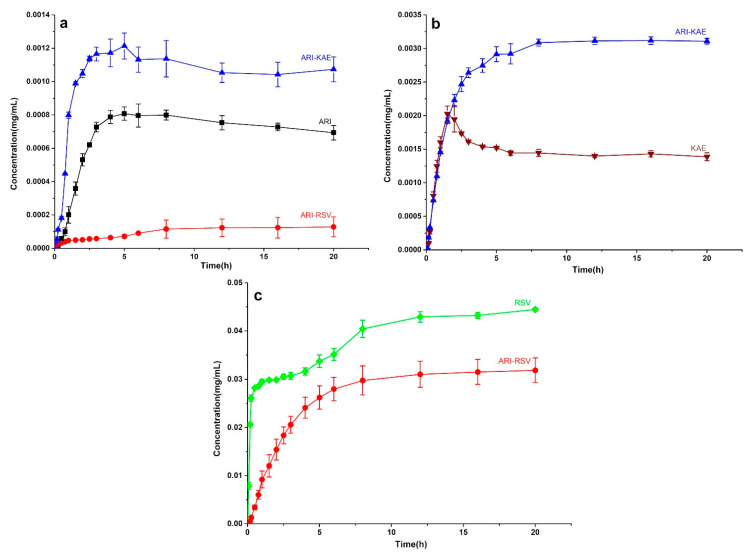
The dissolution profile of ARI-KAE and ARI-RSV at pH = 6.8 phosphate buffer, (**a**) ARI from pure ARI, ARI-RSV, and ARI-KAE, (**b**) KAE from pure KAE and ARI-KAE, (**c**) RSV from pure RSV and ARI-RSV.

The dissolution curves of ARI, RSV, KAE, and their cocrystal in pH = 6.8 phosphate buffer is shown in Figure 12. The result showed that ARI-KAE improves the dissolution of ARI under the condition of pH = 6.8 medium, the dissolution rate is significantly increased, and the solubility is increased for about 50% (Figure 12a). This is due to the endpoint concentration of pure KAE under this condition (1.5 μg/mL) which is twice as high as pure ARI (0.7 μg/mL); however, it is unexpected that the dissolution rate of KAE in ARI-KAE is not reduced, the endpoint concentration is doubled (3 μg/mL) (Figure 12b). The ARI-RSV significantly reduced the dissolution rate of ARI, and the solubility decreased by an order of magnitude; though the endpoint concentration of pure RSV was about 50 times higher than that of pure ARI, but did not increase the dissolution rate of ARI from the cocrystal (Figure 12a). The high concentration of RSV in the system (solubility: 30 μg/mL) limited the dissolution of ARI. The ARI also inhibited the dissolution of RSV (Figure 12c), indicating that RSV and ARI can inhibit each other’s dissolution in pH = 6.8 phosphate buffer.

## 3. Materials and Methods

### 3.1. Materials

Aripiprazole was obtained from Jiangsu Nhwa Pharmaceutical Co., Ltd. (Jiangsu, China), and resveratrol and kaempferol were purchased from Shanghai Macklin Biochemical Co., Ltd. (Shanghai, China). All other reagents were of analytical grade (Shanghai Lingfeng Chemical Reagent Co., Ltd., Shanghai, China) and were used without further purification.

### 3.2. Preparation of Cocrystal

ARI-RSV: liquid-assisted grinding (LAG) was used to prepare ARI-RSV. LAG was performed by manually grinding the ARI powder (224.0 mg, 0.5 mmol) and RSV powder (114.0 mg, 0.5 mmol) in a mortar and pestle for 30 min, and a few drops of acetonitrile were added before starting the manual grinding. The ground product was vacuum dried at 60 °C for 4 h, and, finally, a white powder ARI-RSV was obtained.

ARI_2_-RSV_1_·MeOH: ARI (44.8 mg, 0.1 mmol) and RSV (22.8 mg, 0.1 mmol) were dissolved in 2 mL of mixed solvent of methanol and dichloromethane (3:1, *v*/*v*). The solution was then left at room temperature to slowly evaporate. The solution was filtered by 0.45 μm PTFE filters before evaporating. White flake crystals of the ARI_2_-RSV_1_·MeOH were obtained after 24 h, which were suitable for single crystal X-ray diffraction experiment.

ARI-KAE·EtOH: ARI (44.8 mg, 0.1 mmol) and KAE (28.6 mg, 0.1 mmol) were dissolved in 2 mL of mixed solvent of ethanol and ethyl acetate (1:1, *v*/*v*), and then added 8 mL of anti-solvent n-heptane. Leaving the drug solution at room temperature overnight, ARI-KAE·EtOH were obtained as yellow flaky crystals, which were suitable for single crystal X-ray diffraction experiment. Unfortunately, they were prepared only one time.

ARI-KAE·IPA: 60 mL of isopropanol was heated to dissolve ARI (896 mg, 2 mmol) and KAE (572 mg, 2 mmol), then 60 mL of n-heptane was added and well mixed. The mixture was left overnight to obtain yellow needle-like crystals, which were suitable for single crystal X-ray diffraction experiment.

ARI-KAE: ARI (896 mg, 2 mmol) and KAE (572 mg, 2 mmol) were dissolved in 40 mL of mixed solvent of dichloromethane and tetrahydrofuran (1:1, *v*/*v*), and then 60 mL of n-heptane was added. The mixture was left at room temperature overnight to obtain yellow and small granular crystals.

### 3.3. Characterization Method

#### 3.3.1. Single-Crystal X-ray Diffraction

ARI_2_-RSV_1_·MeOH and ARI-KAE·IPA Single Crystal X-ray Diffraction (SCXRD) data were collected on a Bruker Apex2 CCD diffractometer (graphite monochromated MoKα radiation, λ = 0.71073 Å) (Karlsruhe, Germany) at 296 K. ARI-KAE·EtOH SCXRD data was recorded on XtaLAB PRO diffractometer (graphite monochromated CuKα radiation, λ = 0.154184 Å) (Rigaku, Japan) at 100 K. Data reduction and unit cell refinement were performed with the software SAINT. Crystal structures were solved using direct methods in Shelxs-13 and refined using Shelxl-97 [52]. All non-hydrogen atoms were refined by using full-matrix least squares methods. CIF files can be obtained free of charge from https://www.ccdc.cam.ac.uk/structures (accessed on 24 March 2021) and the Cambridge Crystallographic Data Centre, Cambridge, UK, with the deposition Number 2072710 (ARI_2_-RSV_1_·MeOH), 2072711 (ARI-KAE·EtOH), 2072712 (ARI-KAE·IPA).

#### 3.3.2. Powder X-ray Diffraction

Powder X-ray Diffraction (XPRD) patterns of the samples were collected by using a X’PERT POWDER X-ray diffractometer (PANalytical, Holland) in the θ/2θ scan mode with Cu-Kα radiation (λ = 1.540598 Å). Each X-ray diffractogram was recorded over a 2θ degree of 5° to 40° at a scanning step size of 0.01313° and a scanning rate of 2° per minute. Silicon was used as an external calibrant.

#### 3.3.3. Thermogravimetric Analysis

Netzsch TG 209F3 equipment (Netzsch, Selb, Germany) was used for thermogravimetric analysis (TGA), with a flow of 20 mL/min nitrogen protection, and at a scan rate of 10 K/min from 50 to 200 °C.

#### 3.3.4. Differential Scanning Calorimetry

Netzsch DSC 200F3 equipment (Netzsch, Selb, Germany) was used for differential scanning calorimetry (DSC) analysis, and the sample underwent a heat process (10 K/min from 50 to 300 °C) with a flow rate of 40 mL/min N_2_ as protection atmosphere.

#### 3.3.5. Attenuated Total Reflection Fourier’s Transform Infrared Spectroscopy

A Fourier transform infrared spectrometer (Vertex 70, Brooke Billerica, MA, USA) was used for infrared spectroscopy. In addition, using the attenuated total reflectance (ATR) accessory, the Zn-Se crystal was used to verify the spectrum compared with the sample. The scan range was 400 cm^−1^ to 4000 cm^−1^ with the resolution at 2 cm^−1^.

### 3.4. Powder Dissolution

A dissolution measurement was conducted according to the Chinese pharmacopoeia 2015 edition paddle method on FADT-1202RC automatic sampling dissolution apparatus (Shanghai Fukesi analysis instrument Co., LTD., Shanghai, China). Accurately weighted 60 mg of ARI, 90.5 mg of ARI-RSV, 98.3 mg of ARI-KAE, in triplicate, were milled and passed through 100 mesh sieves. Then, cocrystal and API were added into dissolution vessels that contained 500 mL pH = 4.0 acetate buffer or pH = 6.8 phosphate buffer. The suspension was stirred at 50 rpm (pH = 4.0) or 100 rpm (pH = 6.8) throughout the whole process. The medium temperature was 37 ± 0.5 °C. At 5, 10, 15, 30, 45, 60, 90, 120, 180, 240, 300, 360, 480, 720, 960, and 1200 min, 1 mL of the dissolution samples were withdrawn and replaced by an equal volume of the fresh medium. All the withdraw suspensions were filtered with 0.22 μm nylon filter before high-performance liquid chromatography (HPLC). LC-20AD HPLC (Shimadazu, Kyoto, Japan) was used to analyze the concentration of ARI. The Agilent XDB-C18 column (250 × 4.6 mm, 5 μm) was used for separation and analysis, with a column temperature at 35 °C and UV detection wavelength at 215 nm. The mobile phase was a mixture of acetonitrile and 0.02 M potassium dihydrogen phosphate water (40:60, *v*/*v*). The pH of the potassium dihydrogen phosphate buffer was adjusted to 2.5 with phosphoric acid. The isocratic elution was 15 min, with a flow rate 0.8 mL/min.

## 4. Conclusions

Cocrystal of aripiprazole (ARI) with resveratrol (RSV) and kaempferol (KAE) were synthesized by the solvent volatilization method, anti-solvent method, and liquid-assisted grinding method. The five cocrystals were characterized by using various methods. The cocrystals ARI_2_-RSV_1_·MeOH, ARI-KAE·EtOH, and ARI-KAE·IPA were crystallized in the Pn, P-1, and P-1 space groups, respectively. There are not only hydrogen bonds but also halogen bonds in the ARI_2_-RSV_1_·MeOH crystal, and the ARI tri-molecular motif is unique, which was not found in the reported API crystals. ARI-KAE·EtOH and ARI-KAE·IPA have almost the same tetramers motif; the only difference lies in the way the solvated molecules are connected.

Due to the mutual dissolution inhibition effect of ARI and RSV, ARI-RSV significantly inhibited the dissolution of both components. ARI-KAE tunes the dissolution rate of ARI at different pH medium, while the dissolution rate of the KAE form ARI-KAE is stable, and KAE acts more like a buffer. Although the buffering effect of the dissolution rate is not notable, it still has enlightening significance.

Traditional drug cocrystal design strategies mostly focus on solving the solubility of insoluble drugs, or improving drug stability, permeability, and other physicochemical properties. In this work, we use the basic principles of drug cocrystals to design special drug cocrystals to tune the dissolution behavior of the drug (including reducing the dissolution rate) and provide new design ideas of cocrystal for different routes of administration.

## Figures and Tables

**Table 1:** Data Collection and Refinement Parameters for the cocrystals.

Compounds	ARI_2_-RSV_1_·MeOH	ARI-KAE·EtOH	ARI-KAE·IPA
Empirical formula	C_61_H_70_Cl_4_N_6_O_8_	C_40_H_43_Cl_2_N_3_O_9_	C_41_H_45_Cl_2_N_3_O_9_
Formula weight	1157.03	780.67	794.7
Temperature/K	296.15	100.00 (10)	296.15
Crystal system	monoclinic	triclinic	triclinic
Space group	Pn	P-1	P-1
a/Å	14.837 (4)	11.1969 (2)	9.219 (3)
b/Å	10.201 (2)	13.9621 (3)	10.708 (4)
c/Å	21.184 (5)	14.0547 (3)	20.176 (7)
α/°	90	61.715 (2)	94.863 (5)
β/°	109.556 (3)	86.352 (2)	92.821 (4)
γ/°	90	79.081 (2)	97.687 (5)
Volume/Å^3^	3021.3 (12)	1898.96 (7)	1963.0 (11)
Z	2	2	2
ρ_calc_g/cm^3^	1.272	1.365	1.344
μ/mm^−1^	0.254	2.039	0.225
Radiation	MoKα (λ = 0.71073)	CuKα (λ = 1.54184)	MoKα (λ = 0.71073)
2θ/°	3.992 to 55.138	7.146 to 147.818	3.854 to 54.746
Goodness-of-fit on F^2^	1.032	1.054	1.031
Final R indexes [I ≥ 2σ (I)]	R_1_ = 0.0799, wR_2_ = 0.1792	R_1_ = 0.0438, wR_2_ = 0.1233	R_1_ = 0.0498, wR_2_ = 0.1225
Final R indexes [all data]	R_1_ = 0.1366, wR_2_ = 0.2097	R_1_ = 0.0451, wR_2_ = 0.1244	R_1_ = 0.0740, wR_2_ = 0.1385
CCDC NO.	2072710	2072711	2072712

**Table 2:** Hydrogen bonding table for the cocrystals.

Compounds	D-H⋯A	d(D-H)/Å	d(H⋯A)/Å	d(D⋯A)/Å	∠D-H⋯A/°	Symmetry Code
ARI_2_-RSV_1_·MeOH	O5-H5A⋯N5	0.82	1.93	2.702(9)	157.1	
	O6-H6B⋯O4	0.82	1.98	2.765(8)	161.4	−3/2 + X, 1 − Y, −1/2 + Z
	O7-H7⋯N	0.82	1.99	2.660(9)	138.9	−1 + X, + Y, + Z
	O8A-H8AA⋯O7	0.82	2.07	2.89(2)	176	−1 + X, + Y, + Z
	N3-H3⋯O4	0.86	2.02	2.884(8)	177.8	−2 + X, + Y, −1 + Z
	N6-H6A⋯O2	0.86	2	2.863(9)	177.2	2 + X, + Y,1 + Z
ARI-KAE·EtOH	O2-H2A⋯O9	0.82	1.88	2.6801(18)	166.6	−1 + X, 1 + Y, −1 + Z
	O3-H3⋯O7	0.82	1.87	2.6755(19)	168	−X, 1 − Y, 1 − Z
	O6-H6⋯O9	0.82	2.05	2.7633(16)	145.1	1 − X, 1 − Y, 1 − Z
	O7-H7A⋯N3	0.82	2.02	2.813(2)	163.8	
	O8-H8⋯O4	0.82	1.91	2.6325(16)	147.1	
	N1-H1⋯O4	0.86	1.95	2.7728(18)	160.1	1 − X, 1 − Y, 1 − Z
ARI-KAE·IPA	N3-H3⋯O6	0.86	1.98	2.839(2)	172.3	1 + X, +Y, +Z
	O9-H9⋯N2	0.82	1.96	2.782(3)	175.2	2 − X, 1 − Y, 1 − Z
	O3-H3A⋯O9	0.82	1.82	2.631(2)	172.5	
	O4-H4A⋯O6	0.82	1.9	2.628(2)	147.5	
	O7-H7⋯O2	0.82	1.99	2.691(2)	143.5	−1 + X, +Y, +Z
	O8-H8⋯O2	0.82	1.9	2.711(2)	170.3	3 − X, 2 − Y, 2 − Z

## Data Availability

Date of the compounds are available from the authors.

## Supplementary Materials

The following are available online, Figure S1: Experimental and calculated PXRD patterns of cocrystals.

## Sample Availability

Samples of the compounds are available from the authors.
