# Supplementary material for: New Cocrystals of Antipsychotic Drug Aripiprazole: Decreasing the Dissolution through Cocrystallization
Source: Molecules. 2021 Apr 21;26(9):2414. doi: 10.3390/molecules26092414 (PMC8122301; doi:10.3390/molecules26092414)
Supplement: Supplementary file 1 [file molecules-26-02414-s001.zip › molecules-1176299-supplementary.pdf]

Supplementary Materials

New cocrystals of antipsychotic drug aripiprazole: decrease the dissolution through cocrystallization

Wenwen Liu [1], Ru Ma [1], Feifei Liang [1], Chenxin Duan [1], Guisen Zhang[1,2], Yin Chen[1,*] and Chao Hao[1,2,*]

1 Jiangsu Key Laboratory of Marine Biological Resources and Environment, Jiangsu Key Laboratory of Marine Pharmaceutical Compound Screening, School of Pharmacy, Jiangsu Ocean University, Lianyungang 222005, China; 2019220322@jou.edu.cn (W.L.); 2020000031@jou.edu.cn (M.R.); ylwbs1028@163.com (F.L.); 2019220308@jou.edu.cn (C.D.) gszhang@hust.edu.cn (G.Z.)

2 Department of Biomedical Engineering, College of Life Science and Technology, Huazhong University of Science and Technology, Wuhan 430074, China;

* Correspondence: 2019000015@jou.edu.cn; D201880507@hust.edu.cn; Tel: +86-27-87792235

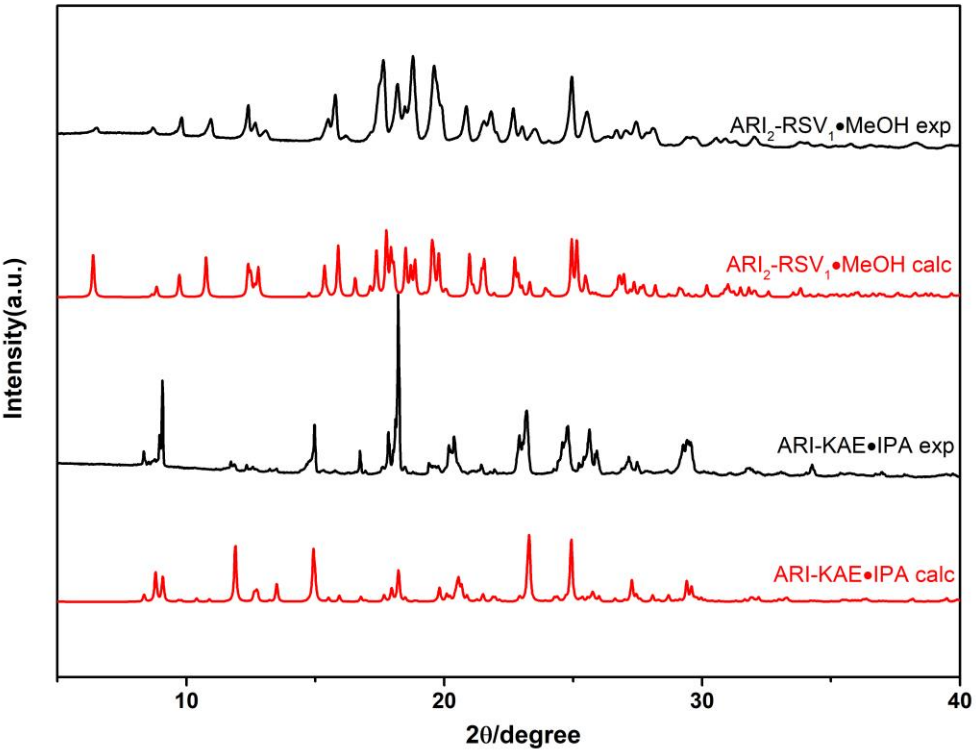

Figure S1. Experimental and calculated PXRD patterns of cocrystals.
